# Working Memory and Executive Function Decline across Normal Aging, Mild Cognitive Impairment, and Alzheimer's Disease

**DOI:** 10.1155/2015/748212

**Published:** 2015-10-15

**Authors:** Anna-Mariya Kirova, Rebecca B. Bays, Sarita Lagalwar

**Affiliations:** ^1^Department of Psychology, Skidmore College, 815 North Broadway, Saratoga Springs, NY 12866, USA; ^2^Neuroscience Program, Skidmore College, 815 North Broadway, Saratoga Springs, NY 12866, USA

## Abstract

Alzheimer's disease (AD) is a progressive neurodegenerative disease marked by deficits in episodic memory, working memory (WM), and executive function. Examples of executive dysfunction in AD include poor selective and divided attention, failed inhibition of interfering stimuli, and poor manipulation skills. Although episodic deficits during disease progression have been widely studied and are the benchmark of a probable AD diagnosis, more recent research has investigated WM and executive function decline during mild cognitive impairment (MCI), also referred to as the preclinical stage of AD. MCI is a critical period during which cognitive restructuring and neuroplasticity such as compensation still occur; therefore, cognitive therapies could have a beneficial effect on decreasing the likelihood of AD progression during MCI. Monitoring performance on working memory and executive function tasks to track cognitive function may signal progression from normal cognition to MCI to AD. The present review tracks WM decline through normal aging, MCI, and AD to highlight the behavioral and neurological differences that distinguish these three stages in an effort to guide future research on MCI diagnosis, cognitive therapy, and AD prevention.

## 1. Introduction

Alzheimer's disease (AD) is the sixth leading cause of death in the United States and the fifth leading cause of death in people over the age of 65, as determined by the Center for Disease Control [1]. The risk of developing AD increases exponentially with age [2]. Currently, verification of an AD diagnosis occurs through postmortem detection of pathology in neural tissue, specifically extracellular amyloid plaques and intracellular neurofibrillary tau tangles; however, cognitive changes are discernible early during AD pathogenesis and mild cognitive impairment (MCI). The following review examines the detection of working memory (WM) deficits through behavioral, functional, and structural changes amongst nonimpaired, MCI, and AD adults. We further investigate if tracking WM and executive function skills over time, in addition to biomarker analysis, can identify individuals during MCI as being at risk for progression towards AD.

## 2. Tracking Decline Pre- and Post-AD Onset

Between 10 and 20 percent of adults above the age of 65 are diagnosed with MCI [3–5], and approximately 10 percent of MCI adults progress to AD [6]. Compared to nonimpaired age-matched adults, those with MCI tend to develop AD more rapidly [7]. Neuropathologically, MCI adults exhibit amyloid plaques and neurofibrillary tau tangles in AD-vulnerable regions of the brain responsible for episodic memory, specifically in the olfactory cortex, subiculum, and parahippocampal gyrus in the medial temporal lobes [8, 9], albeit to a lesser degree compared to an AD patient's brain [10].

Behavioral research indicates that MCI adults show cognitive deficits in WM, central executive function, and attentional resources compared to nonimpaired age-matched controls [11–15]. Preclinical individuals self-report subjective cognitive decline prior to evidence of impairment on cognitive assessments [16]. While WM impairments during MCI fall short of measurable interference with activities of daily living, they are more severe than WM deficits that result from normal aging. Although these impairments do not involve episodic memory, they can reliably predict the progression from MCI to AD [16, 17], particularly when paired with deficits in episodic memory [13, 15]. Taken together, results of neuropathology and cognitive impairment suggest that AD diagnosis can be based on a continuum that begins as MCI and develops into AD, although there may be more subtle signs prior to an MCI diagnosis.

These observations raise two unanswered questions: (1)* what differences exist between cognitively normal, MCI, and AD adults regarding neural activation and recruitment of WM resources*? and (2)* what can these differences tell us about an individual's likelihood of progressing to MCI or AD*? Research suggests there is great potential for cognitive deficit reversal and strengthening of executive function at the MCI stage [18]. MCI adults are reported to regain cognitive function by engaging in physical exercise [19], eating healthy foods (e.g., fish oils) [20], reducing LDL cholesterol intake [21], and practicing challenging cognitive tasks [22]. Therefore, clinical tools that accurately detect the progression from normal cognitive aging to MCI award preclinical adults the opportunity to actively participate in tasks that may help improve and preserve their cognitive function.

## 3. Working Memory: A Brief Overview

WM is a system that underpins cognitive activities ranging from attention allocation to specific stimuli (i.e., selective attention) to complex decision-making [23]. More specifically, WM promotes active short-term maintenance of information for later access and manipulation [24]. The form of the information held in WM is both auditory, as maintained by the phonological loop (e.g., to promote language comprehension), and visual, as maintained by the visuospatial sketchpad (e.g., to promote visuospatial reasoning). Behavioral methods reveal that each of these subsystems is controlled by the central executive, an attentionally limited gatekeeper that selects auditory and visual material for maintenance and manipulation [24]. Importantly, the central executive is not a memory system but an attention controller. Because attention span is limited and subject to individual differences, executive functioning varies with normal aging. For example, nonimpaired older adults exhibit impairments associated with the number of items they can selectively hold in a subsystem at any given time, suggesting WM load impairment [25, 26].

WM capacity may be assessed using behavioral tasks that result in a quantitative measure of memory span. These tasks require participants to encode lists of stimuli (such as letters, digits, words, or pictures), often involving either a selective attention task during encoding (i.e., attending to just one feature of a stimulus, such as the font color of the letters) or a divided attention task (i.e., attending to multiple features of a stimulus, such as font color and location on a computer screen) to further tax WM capacity [24]. As one example of a span task, in the “*n*-back task,” participants monitor a series of presented stimuli during encoding and are asked to recall whether a specific stimulus matches one presented *n* trial previously, typically 1, 2, or 3 [27]. The behavioral measure of WM capacity is the number of correctly matched stimuli. Tasks such as the *n*-back task require divided attention and online monitoring, a process of updating the memory of presented stimuli which taxes the central executive's limited resources [27].

Region-specific neural activation in response to a memory task is analyzed through neuroimaging techniques including functional magnetic resonance imaging (fMRI) and positron emission tomography (PET). These neuroimaging methods depict recruitment of specific brain regions during a WM task, demonstrating that activation of the prefrontal cortex (PFC), parietal regions, cingulate gyrus, and hippocampus are associated with WM processing in nonimpaired young adults [28]. To return to the *n*-back task as one measure of WM, neuroimaging during the completion of that task reveals activation of the posterior parietal cortex, premotor cortex, rostral PFC, dorsolateral PFC, and ventrolateral PFC [27]. These neuroimaging tools allow researchers to compare brain activity during WM tasks of different populations to uncover similarities and contrasts between the selected groups.

## 4. Working Memory in Normal Aging

One model addressing neural activation differences between nonimpaired older adults (typically 60+ years old) and nonimpaired younger adults (typically 18–30 years old) during WM tasks is the Hemispheric Asymmetry Reduction in Older Age model, or HAROLD [29]. Findings in support of the HAROLD model reveal that, during WM tasks, young adults display left PFC activation during verbal WM tasks and right PFC activation during spatial WM tasks, while older adults display bilateral activation of the PFC during both verbal and spatial WM tasks [30]. Initially, this lack of asymmetrical, specialized activation in older adults was believed to indicate poor cognitive function and inefficient communication between the two hemispheres, as older adults were not recruiting the correct resources. However, behavioral and neuroimaging data support a* compensatory view* of bilateral activation where older adults compensate for age-related decline by recruiting additional neural networks to maintain performance on the task at lower levels of complexity. These tasks involve fewer stimuli for WM maintenance and do not require as many attentional resources as more complex task levels [31]. Further, when older adults receive environmental support during complex task performance, brain region recruitment mirrors that of younger adults. For example, older adults display enhanced recruitment of the left frontal cortex, almost to the same degree as young adults, when executing semantic encoding tasks while receiving semantic elaboration prompts from an experimenter [32]. Frontal cortex recruitment suggests that the frontal cortical resources are functioning in older adults but are not recruited properly during self-initiated tasks requiring executive control.

Expounding on the compensation view of the HAROLD model, the Compensation-Related Utilization of Neural Circuits Hypothesis (CRUNCH) [33] asserts that overactivation of neural circuits in nonimpaired older adults, relative to nonimpaired younger adults, is necessary for completion of WM tasks and correlates with greater performance when normalized for task demand. Additionally, during specific memory tasks (e.g., incidental encoding of complex visual scenes) nonimpaired older adults display bilateral overactivation of the frontal cortex, including the PFC, to compensate for decreased activity with age of more specialized regions such as the medial temporal lobes, relative to nonimpaired younger adults [34]. Payer et al. [35] found that, during a visual WM task in older adults, underactivation of the ventral visual cortical (VVC) pathway, which includes areas of the occipital and temporal lobes, was accompanied by bilateral overactivation of the PFC.

The visual WM task involved encoding and identifying face and house stimuli that typically activate two regions of the VVC pathway: the fusiform face area and the parahippocampal place area, respectively. Twelve younger and 12 older adults were presented with three consecutive images of either faces or houses. Following image presentation, participants viewed a probe image and decided whether the image matched any one of the three preceding target images (an example of an *n*-back task). Young adults matched the probe image stimulus to target stimuli with greater accuracy than older adults, although there was no significant difference in response times between the two age groups. Interestingly, Payer et al.'s [35] imaging data failed to reliably show PFC overactivation in conjunction with VVC pathway underactivation; however, the authors note that there was a trend present. In sum, the neuroimaging data hint at bilateral overactivation as a mechanism for compensation in old age as explained by the CRUNCH model, although older adults still performed poorly compared to younger adults.

Schneider-Garces et al. [36] further assessed the impact of aging on WM by examining whether the patterns of bilateral overactivation in nonimpaired older adults predicted by the CRUNCH model would disappear when task demand was comparable across age groups. Task demand was estimated from individual performance on the Sternberg task [37], a task that requires participants to remember whether a single probe stimulus was part of a series of stimuli presented previously. Neural activation differences between nonimpaired older and younger adults during completion of the Sternberg task can be entirely attributed to WM span differences regardless of age. Further, younger adults exhibited bilateral overactivation of specific cortical areas such as the dorsolateral PFC when task demands were high (4–6 stimuli presented with the probe), suggesting that bilateral activation can be a beneficial mechanism for recruiting additional neural resources earlier in life rather than a sign of cognitive impairment.

Findings from Schneider-Garces et al. [36] further suggest that impaired WM capacity in older adults is due to reduced selective attention. Older adults struggled with Sternberg task completion when the number of stimuli presented exceeded their working memory span; they also displayed slower response times and lower accuracy rates, especially at higher WM loads (4–6 item sets). Older adults could not maintain more than four items in WM at a given time, while younger adults could maintain five or more items in WM. Neurologically, this decrease in capacity and processing speed is associated with age-related changes in the dorsolateral PFC [38].

Both groups in Schneider-Garces et al.'s [36] study exhibited neural activation of the occipital, premotor, parietal, prefrontal, and medial frontal areas during task completion. Younger adults showed mainly left hemispheric activation with some bilateral activation of frontal and parietal regions at high loads, suggesting that increasing task demand typically leads to recruitment of contralateral brain regions as posited by Reuter-Lorenz and Cappell [33] and the CRUNCH model. Older adults, on the other hand, exhibited increased neural activity (overactivation of bilateral brain regions) during low WM loads (2–4 item sets) and underactivation at high loads, suggesting that overactivation occurs only when tasks are easy enough for compensatory mechanisms to be beneficial. Underactivation occurs when the task exceeds WM capacity (4+ items for older adults). The authors speculate that older adults' brain activity peaked during low loads and declined during high loads because a smaller WM span results in decreased attentional control and greater susceptibility to interfering stimuli, a marker of failed inhibition [36]. Interestingly, the authors examined neural activation in relation to each participant's WM span and found no differences in levels of brain activity at respective peak WM capacity across age groups.

## 5. Working Memory in MCI

MCI is often characterized by slight but noticeable deficits in attention, learning and memory, executive function, processing speed, and semantic language [13–15]. Over the years, researchers have adjusted the definition of MCI as well as the cognitive and pathological criteria that define the various subsets of MCI [39, 40]. MCI is divided into three subtypes: amnestic-MCI (a-MCI), multiple domain a-MCI (a-MCI+), and nonamnestic-MCI (na-MCI) [13]. a-MCI adults exhibit objective episodic memory impairments, a-MCI+ adults exhibit episodic memory and other cognitive impairments (e.g., WM, executive function, processing speed, and attentional processing), and na-MCI adults exhibit cognitive impairments not related to episodic memory [13]. It was previously believed that a-MCI had the highest rate of conversion to AD given the profound episodic memory deficits in AD; however, recent findings suggest that early impairments in visual episodic memory, executive function, semantic language/memory, attention, and WM are also strong predictors of progression from MCI to AD [13, 41, 42].

As demonstrated by Schneider-Garces et al. [36], controlling for WM load differences yields a reliable measure of WM capacity that is independent of age. Kochan et al. [12] used task load to assess visuospatial WM in MCI because assessing WM decline in this preclinical population compared to nonimpaired adults may provide insight into how pre-AD pathogenesis affects WM. The researchers recruited 35 MCI individuals with various MCI subtypes and 22 age-matched nonimpaired controls to complete a visuospatial WM task with three load levels (low, medium, and high) while being imaged in an fMRI scanner. During each trial, participants viewed a grid with abstract square shapes, the target stimuli. During the encoding phase of the task participants had to remember the position of each square on the grid, and in the retrieval phase that followed seconds later they had to identify whether there was at least one square that remained in the same encoding trial position. The researchers implemented a calibration method for the WM task to control for each participant's performance level to maintain 75–85% accuracy for the medium-load trials and 60–70% accuracy for the high-load trials.

The behavioral results from Kochan et al. [12] indicated that the percentage of MCI adults receiving lower load stimulus sets compared to higher load sets in order to achieve the calibration criteria was significantly greater than nonimpaired adults. Additionally, MCI participants exhibited slower response rates for the 2-target and 3-target trials compared to the nonimpaired controls, although this difference was only marginally significant. Upon examination of the brain imaging data, nonimpaired age-matched controls displayed increased activation of the right precuneus region of the parietal lobe and the right anterior cingulate gyrus (regions associated with WM) with increasing task load. The reverse was observed for the MCI group, where activation of these regions was greater than the nonimpaired group at low loads (overactivation) and decreased as load increased (underactivation). The MCI group as a whole, regardless of subtype classification, showed WM behavioral and neurological deficits compared to the age-matched control group.

Clément et al.'s [11] research demonstrates that compensation and neuroplasticity occur not just in normal aging but in MCI, too, as lesions and brain damage are not significant enough to hinder the recruitment of additional neural resources needed to accomplish cognitive tasks requiring executive function control (e.g., manipulation and divided attention). The authors proposed that as MCI progressed toward AD there would be a disintegration of the compensatory networks as observed by underactivation of the lateral PFC, precuneus, and posterior parietal regions, all involved in executive function, compared to nonimpaired age-matched controls. Furthermore, the authors hypothesized that MCI participants with higher cognitive functioning would display overactivation of those brain regions relative to nonimpaired controls and that this overactivation would be positively correlated with performance on the two tasks. The 14 a-MCI and a-MCI+ participants were split evenly into two groups, MCI higher-cognition and MCI lower-cognition, based on their scores on the Mattis Dementia Rating Scale (MDRS) [43] assessing global cognitive function. The manipulation task required online monitoring and manipulation of the alphabet while solving alphanumeric equations. For the divided attention task participants were required to solve more alphanumeric equations while monitoring if a color change occurred on the screen. There was a focused attention control task for both the manipulation and divided attention tasks, during which participants looked at a random string of numbers and letters and had to report whenever the font color changed from black to red.

The behavioral results showed that the MCI lower-cognition group was significantly more impaired than the MCI higher-cognition group and the nonimpaired control group on neuropsychological measures of processing speed and executive function. For the manipulation task, the two MCI groups performed significantly worse (i.e., answered fewer questions correctly) than the control group, and there was no significant difference between the two MCI groups. For the divided attention task, the MCI lower-cognition group performed worse than both the MCI higher-cognition group and the nonimpaired control group, and there was no significant difference between the MCI higher-cognition group and the nonimpaired controls.

The neuroimaging data showed that during the manipulation task MCI higher-cognition participants displayed significant overactivation in the left postcentral gyrus and the left middle and superior frontal gyri compared to the nonimpaired controls. The MCI lower-cognition group showed underactivation in the left inferior and middle frontal gyri and left occipitotemporal regions compared to the nonimpaired controls. As for the divided attention task, MCI higher-cognition participants demonstrated overactivation of the left inferior frontal gyrus, the left insula, the left caudate and putamen, the left thalamus, left cerebellum and midbrain, left fusiform gyrus, and the left and right anterior cingulate cortex. For the MCI higher-cognition group increased performance on the divided attention task was directly correlated with greater activation of the right putamen, the anterior cingulate cortex (responsible for WM), the left caudate, the left insula, and the left inferior frontal gyrus [11].

Clément et al. [11] claim that the data support their hypothesis that greater activation is indicative of neuroplasticity, which compensates for cognitive decline in the very early stages of MCI when individuals still have relatively intact cognitive abilities. This compensation is evident from the positive correlation between brain activation and performance on the divided attention task for the MCI higher-cognition group. On the other hand, performance on the manipulation task was not positively correlated with the observed overactivation in MCI higher-cognition participants. These inconsistencies could be a sign that manipulation capabilities start to deteriorate earlier than divided attention, but more research on these two attentional processes is needed using different assessment tools to clarify the existing discrepancies. Based on these findings, tracking executive function performance with divided attention and manipulation tasks in older adults can be a useful tool to assess an individual's cognitive abilities as one ages.

## 6. Factors Predicting Progression from MCI to AD

In order to examine when executive function and WM deficits appear, Belleville and colleagues [16] compared attentional control performance using tasks to assess divided attention, online manipulation of stimuli, and the ability to inhibit unrelated stimuli among participants in a-MCI, a-MCI+, and mild early-stage AD. Manipulation skills were assessed using an alphabetical recall task [44, 45], inhibition of interfering stimuli was assessed with the Hayling task [46], and divided attention was assessed using the adapted Brown-Peterson procedure [45]. AD participants performed significantly worse than the nonimpaired age-matched adults on all three tasks of attentional control, whereas MCI participants performed worse than nonimpaired controls just on the Brown-Peterson procedure when there was a 30-second delayed recall.

One year following the study, a subset of the MCI participants was assessed for progression to AD. Eight people were diagnosed with AD, leading authors to revisit the data to determine if there were factors that predicted their AD prognosis. Belleville et al. [16] reported that those MCI participants who later declined to AD performed significantly worse than the control group on measures of manipulation skills and divided attention. The Brown-Peterson procedure proved to be the most sensitive of the three assessments, suggesting that deficits in divided attention may be one of the first signs of WM decline in the preclinical stages of AD. These deficits in divided attention may appear around the same time that episodic memory deficits emerge. The attentional control deficits that quickly follow as individuals with MCI progress to AD include manipulation skills and failed inhibition of irrelevant stimuli. The results indicate that individual scores on divided attention and manipulation skills seem to be predictors of progression from MCI to AD.

In light of recent research suggesting the importance of deficits in attention, WM, and episodic memory in predicting the conversion of MCI to AD, Summers and Saunders [15] designed a longitudinal study to track which MCI subtypes were more likely to progress to AD over a 20-month period; this study was an extension of a previous longitudinal study by Saunders and Summers [13]. Researchers administered a series of clinical and neuropsychological tests assessing verbal and visual episodic memory, WM, attention, executive function, and language processing at the start of the study and 20 months after study commencement. At the 20-month assessment, 10 of the 81 MCI participants (12%) were determined to have progressed to AD and 20 participants (25%) regained normal cognitive function and memory. Only participants with a baseline diagnosis of a-MCI+ were identified as having “probable AD” at 20 months by a physician who was blind to the participants' initial diagnosis. The remaining 51 participants (63%) continued to meet MCI criteria, and 25% of the 12 participants initially diagnosed with a-MCI progressed to a-MCI+. Researchers point to the need for more sensitive diagnostic criteria for MCI and for predicting which MCI individuals will progress to AD by means of longitudinally assessing cognitive function and testing for biomarkers for AD [15, 39, 40].

In agreement with Summers and Saunders [13], Klekociuk et al. [42] argue that the current MCI diagnostic tools are not sensitive enough, thus leading to great variability in the number of MCI individuals who regain normal levels of function over time and those who remain to be diagnosed with MCI. The researchers utilized a mix of cognitive and neuropsychological assessments to better predict which participants had MCI and would most likely progress to AD. The battery of neuropsychological tests used at screening was not the same as the battery of assessments used at the 9-month and at 20-month poststudies, though the same categories of function were assessed. A robust set of tests assessing nonmemory components were administered throughout the study. The results indicate that tests of sustained attention, semantic memory, WM, episodic memory, and selective attention led to correct predictions of MCI or unimpaired function with 80% accuracy. These assessments can also be used in clinical settings to monitor cognitive function and serve as a red flag for worsening performance on executive function and WM tasks over time, especially in conjunction with worsening episodic memory. The authors suggest that the a-MCI+ subtype is the only true form of MCI because declines in episodic memory and cognitive function together are distinctly associated with MCI.

The present literature has not conclusively shown whether WM, specifically executive function, predicts the progression towards AD. For example, a fairly recent study by Peters et al. [47] examined neuropsychological tests and brain imaging data to determine which factors accounted for progression from MCI to AD. Of importance is the fact that the authors did not account for different MCI subtypes. The participants in the 2014 study were diagnosed with MCI based on criteria established previously by Petersen and Morris [48]: (1) participant complaints of cognitive or memory deficits; (2) a score of 1.5 standard deviations below average (based on age and level of education) on measures assessing episodic memory, language, or attention; (3) no functional impairment with activities of daily living; and (4) a score of 26 or below on the Mini Mental Status Exam (MMSE) [49] and 130 or below on the MDRS [43] which are the two major assessments of global cognitive function. As argued by Klekociuk et al. [42], the MMSE is not sensitive in diagnosing MCI and produces a high false-positive rate. Consequently, the screening criteria used by Peters et al. [47] are too broad to distinguish between the three MCI subtypes, and as a result correlations between AD onset and specific MCI subtypes cannot be distinguished in this study.

Participants completed neuropsychological and brain imaging tests three times during a two-year period to determine which participants progressed to AD [47]. At the two-year mark 18 of the 40 total MCI participants (45%) progressed to dementia; of those, three (17%) were diagnosed with mixed dementia (i.e., met criteria for both probable AD and vascular dementia) and 15 (83%) were diagnosed with probable AD. The imaging results showed that progression from MCI to AD could be predicted by observing structural brain changes, specifically cortical thinning of the right anterior cingulate and middle frontal gyri. The neuropsychological results reported that poor performance of episodic memory and free recall, but not of executive function and WM, predicted progression to AD. However, the right anterior cingulate and middle frontal gyri are associated with WM, specifically with selection and inhibition, processes by which individuals choose relevant information to remember and irrelevant information to discard [50]. The discrepancy between the imaging and neuropsychological data in Peters et al.'s [47] study could be due to the type of WM assessment which specifically measured task switching and planning but not inhibition. Thus the limited neuropsychological tests do not tell the full story behind the observed structural changes. Furthermore, the authors note the small sample size of 40 MCI participants.

## 7. Working Memory in AD

Baudic et al. [51] examined executive functioning skills in adults with AD to determine when executive function deficits emerged as the disease progressed. Participants with very mild and mild AD exhibited executive function deficits relative to nondemented control subjects as determined by their scores on a verbal fluency test, Raven's Colored Progressive Matrices [52] testing visuospatial abilities, Mental Control task (naming the months backwards) [53], Trail Making Test, Part B [54], assessing executive function, and a Modified Card Sorting Test [55] assessing perseveration. The observed deficits in visuospatial skills were explained in terms of executive functioning through poor decision-making. Although there was no significant difference between the two AD populations in performance on the Mental Control task, the mild AD patients (but not the very mild AD patients) performed worse than nonimpaired controls on the Mental Control task. Additionally, both AD groups performed significantly worse than nondemented controls on the Trail Making Test and the Modified Card Sorting Test. As a whole the results support the idea of persisting executive function deficits and perseveration in early-stage mild AD.

Castel and colleagues [56] examined two components of WM in AD by measuring WM efficiency (the number value of the words recalled) and capacity (number of words recalled). To measure WM efficiency, Castel et al. [56] assigned a value from 1 to 12 to words that participants were required to memorize from a list. Participants with early-stage AD (mild and very mild) and nonimpaired younger and older adults were instructed to memorize words with the highest point value to maximize their score. The results indicated that young adults recalled significantly more words than all other groups, thereby demonstrating greater WM capacity. The younger and older nonimpaired adults earned significantly greater value scores (a sign of WM efficiency) than both of the early AD groups. This pattern was present even when controlling for recall performance (a measure of WM capacity), thus indicating deficits in selective attention in the AD population.

## 8. Therapeutic Approaches and Cognitive Tasks to Activate WM

Researchers have learned much about WM decline in normal aging over the recent years. Reuter-Lorenz and Park [57] suggest that aging puts a strain on WM and that the brain must recruit additional contralateral resources even at low task demand to compensate for the decline (for a review of their Scaffolding Theory of Aging and Cognition, STAC, see [57]). Identification of the functional, structural, and behavioral hallmarks of normal aging aids in our understanding of atypical changes indicative of dementia. There is some research to suggest that this cognitive restructuring occurs in the MCI brain too, before the pathology has caused further brain damage, as in the case of AD [11]. The* underrecruitment theory* [32] suggests that the frontal brain regions may be viable but not voluntarily activated during encoding and retrieval in MCI. This calls for intervention strategies to strengthen neural pathways and preserve cognitive abilities, especially WM and executive function, in MCI. Based on the current literature reviewed, the interventions should target selective and divided attention, manipulation skills, and inhibition of interfering stimuli. These cognitive functions as part of the central executive are essential for WM efficiency and maximizing capacity; they are compromised early in AD and even during MCI. The current research on therapeutic approaches in MCI is encouraging but results are inconsistent across studies due to varying methodologies, small sample sizes, and lack of long-term follow-up.

Li et al. [58] conducted a meta-analysis of 17 studies assessing the effects of cognitive stimulation or cognitive rehabilitation on MCI participants. The results indicated that while the nonimpaired controls did not improve on measures of global cognition (MMSE scores), episodic memory, executive function/WM, visuospatial processing, or attention/processing speed, the MCI participants receiving some form of cognitive intervention made significant gains in episodic memory and executive function/WM relative to the MCI controls. Some of the interventions focused on attention, executive control, auditory processing speed and auditory WM, practical problem solving, stress reduction techniques, and occupational therapy.

A theoretical rehabilitation model for MCI proposed by Huckans et al. [59] shows how identified protective factors (Mediterranean diet and physical/mental exercise) and risk factors (smoking and heavy alcohol consumption) mediate the progression to dementia or reversal to normal cognition. The authors reviewed multiple studies of four nonpharmacological cognitive rehabilitation therapies (CRTs) used to promote reversal to normal cognitive functioning and decrease risks of progression to AD: restorative cognitive training, compensatory cognitive training, lifestyle interventions, and psychotherapeutic interventions. As a whole, the studies failed to consistently show the beneficial effects of CRTs, but some of the reviewed articles implemented a stronger experimental design and their results reflect the positive impacts of CRTs on MCI. For example, Scherder et al. [60] reported that aerobic exercise for at least 30 minutes daily (3 times a week) helped improve executive control. Additional CRT studies examining lifestyle interventions reported that resistance training twice a week for a 12-month period helped improve executive functioning, memory, and selective attention in MCI adults [61]. A study by Tsolaki et al. [62], which featured the largest sample size, implemented a multimodal CRT program in which MCI participants took part in sixty 1-hour sessions over a period of 6 months focusing on attention, memory, and executive function in addition to psychotherapeutic treatment. The MCI participants in the CRT group significantly improved on measures of global cognition, memory, attention, visuomotor ability, executive function, language, and daily functioning between pre- and postassessment. These studies, reviewed in depth in Huckans et al. [59], demonstrate that neuroplasticity and cognitive restructuring in the MCI brain can decrease the risk of developing AD.

## 9. Conclusions

Recent research has illuminated compensatory mechanisms that occur in the brain as a result of aging. Older adults recruit bilateral regions of the PFC to complete WM tasks requiring executive control. Overactivation of bilateral PFC regions in older adults is an example of cognitive restructuring as individuals begin to experience difficulties with executive function tasks such as divided attention and inhibition of interfering stimuli. As a result older adults have a lower WM capacity than younger adults.

Executive dysfunction becomes more pronounced during MCI, and an increasing number of studies have reported the existence of both cognitive and memory deficits in MCI. Brain imaging data also demonstrate that MCI individuals display underactivation compared to nonimpaired adults on WM tasks of increasing load. This evidence promotes the use of WM and executive function assessments to track behavioral and functional changes to distinguish between normal aging, MCI, and AD. One suggestion is to implement a robust set of neuropsychological tests into clinical practice which detect WM and executive functioning deficits over time. Follow-up on WM and executive function skills over time can pinpoint stages of cognitive decline and prompt for cognitive intervention during the preclinical stages of AD when interventions are likely to have the greatest effect on cognitive functioning.

Importantly, very early stages of AD are marked by executive dysfunction and WM impairments in addition to episodic memory deficits. These cognitive deficits begin during MCI and appear to be a sign of progression to AD. Therefore, future studies are recommended to detect and monitor changes in WM, attention, and executive function in MCI and older nonimpaired individuals. The critical preclinical period also provides the opportunity for cognitive interventions to stop or slow the progression to AD. Such studies on therapeutic interventions should focus on tasks that emphasize executive function skills like selective or divided attention, inhibition, manipulation, and task switching. Incorporation of assessments and interventions that are targeted towards WM, attention, and executive function in research and clinical settings expands the tools available to monitor and treat patients at the early stage of disease and provide the benefit of being of low cost, noninvasive, and relatively easy to implement.
